# Modulation of Mechanical Control of Relaxation in HFpEF: Investigating Levosimendan’s Effects on Cardiac Relaxation

**DOI:** 10.51894/001c.143920

**Published:** 2025-09-30

**Authors:** Jia Yi Koh, Anita Abbo, Charles S. Chung

**Affiliations:** 1 College of Osteopathic Medicine Michigan State University; 2 Wayne State University

## 17

### INTRODUCTION

Nearly 7 million persons are diagnosed with heart failure in the USA. More than half are diagnosed with heart failure with preserved ejection fraction (HFpEF), a complex clinical syndrome characterized by impaired left ventricular relaxation and increased myocardial stiffness. Five-year mortality remains above 50% and no clear treatment has emerged. Our lab has developed and studied Mechanical Control of Relaxation (MCR), the strain rate dependence of the relaxation rate of myocardial tissue, independent of afterload. To understand mechanism and screen for therapies that might enhance MCR, and thus treat HFpEF, our lab previously investigated how modulating myosin using Omecamtiv Mecarbil (OM) affects MCR. OM, a myosin activator (myotrope), increases MCR, but the study identified that non-myosin mechanisms may be important (Cavanaugh, MSUCOM Class of 2025).

### OBJECTIVES

In this study, we examined whether thin filament regulation modifies MCR. Levosimendan (LEVO) is a calcium sensitizer that binds to cardiac troponin C and enhances myocardial contractility. We hypothesize that through LEVO’s mechanism in increasing calcium sensitivity, it will increase isometric force as well as MCR.

### METHODS

Intact cardiac trabeculae were isolated from rat hearts, superfused, and electrically stimulated (paced) at their optimal length (Lo). Contractility was determined during isometric twitch contractions. MCR was determined by varying the end systolic strain rate during phase that mimics ejection. LEVO was added at increasing concentrations to determine its effects on contraction and relaxation

### RESULTS

Preliminary data indicate that increasing LEVO dose correlates with higher isometric contraction force and faster isometric relaxation. However, no clear changes in MCR were observed.

### CONCLUSION

Troponin-modulating drugs influence both isometric contractility and relaxation rates, offering a potential therapeutic avenue for improving diastolic function in HFpEF. However, further data collection is needed to determine LEVO’s specific effects on MCR.

